# The Mediating Role of Academic Resilience and Cognitive Test Anxiety in the Association Between Smartphone Addiction and Academic Achievement

**DOI:** 10.1002/brb3.70800

**Published:** 2025-09-01

**Authors:** Hakan Koğar, Ayfer Sayın, Güçlü Şekercioğlu, Esin Yılmaz Koğar, Hüseyin Kafes

**Affiliations:** ^1^ Faculty of Education Akdeniz University Antalya Turkey; ^2^ Faculty of Education Gazi University Ankara Turkey; ^3^ Faculty of Education Niğde Ömer Halisdemir University Niğde Turkey

## Abstract

**Objectives:**

Smartphone addiction is the loss of self‐control resulting from excessive use of smartphones and is the most challenging addiction to daily life among technology addictions. Some studies explain the relationship between smartphone addiction and academic achievement. The purpose of this research is to examine the mediating effect of academic resilience, test anxiety, and general self‐efficacy in the association between academic achievement and smartphone addiction.

**Methods:**

A total of 828 seventh‐ and eighth‐grade students from seven different cities in Turkey participated in this study. To test the theoretical model proposed, the PROCESS macro for SPSS was performed to examine the chain mediation model (model 6) using 10,000 bootstrapping.

**Results:**

The effect of smartphone addiction on academic achievement is negative and statistically significant. General self‐efficacy did not play a mediation role in the relationship between smartphone addiction and achievement in school, but academic resilience and cognitive test anxiety did.

**Conclusions:**

Cognitive test anxiety and academic resilience mediated the relationship between smartphone addiction and academic achievement. It is thought that these findings will guide teachers and school administrators trying to increase academic achievement.

## Graphical abstract

The direct effect of smartphone addiction on academic achievement is statistically significant (*B* = ‐0.118, 95% CI [‐0.219, ‐0.017], *p* < 0.05). The total effect obtained when the effect of mediating variables is included is also statistically significant (*B* = ‐0.274, 95% CI [‐0.370, ‐0.177], *p* < 0.001). Cognitive test anxiety and academic resilience mediated the relationship between smartphone addiction and academic achievement. General self‐efficacy was excluded from the model since it did not have an effective mediating role.

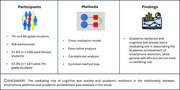

## 1 Introduction

The transition of students from secondary to high school education can be very stressful. One of the main sources of this stress is some centralized high‐stakes tests. These centralized tests, along with in‐class tests, can create stress on students and lead to some maladaptive academic behaviors by limiting their academic decision‐making processes (Deb et al. 2015; Pascoe et al. 2020). This can lead to a variety of different psychological difficulties, such as increased test anxiety and decreased academic resilience. Problems with self‐efficacy may also arise in children due to the presence of a centralized exam and the expectations of the family and social environment about success. It may be inevitable for children who experience socialization and self‐efficacy problems due to exam anxiety and academic expectations to face the problem of smartphone addiction (Chiu 2014; Zhou et al. 2020). For these reasons, this study aims to examine the effects of self‐efficacy, smartphone addiction, test anxiety, and academic resilience on academic achievement.

### 1.1 Smartphone Addiction and Academic Achievement

Smartphone addiction is the loss of self‐control resulting from excessive use of smartphones and is the most challenging addiction to daily life among technology addictions (Liu 2021). Significant correlations between smartphone addiction and negative psychological outcomes such as anxiety (Fekih‐Romdhane et al. 2023), stress (Gökçearslan et al. 2018), and depression (Stanković et al. 2021) have been reported. Many students reported that they overuse smartphones even at school and that smartphone use distracts them from studying, suggesting that overuse of smartphones is a factor that can prevent students from focusing on academic work (Lin et al., 2014). However, there are very few studies explaining the relationship between smartphone addiction and academic achievement. The findings of regression analysis conducted in one of these studies, smartphone addiction of elementary school students, explained academic achievement statistically significantly (Eoh et al. 2022). Other studies have also found a negative correlation between smartphone addiction and academic achievement (Lepp et al. 2014; Li et al. 2015). However, both studies focused on college students. For these reasons, there is a need for studies on how much and how smartphone addiction affects the academic achievement of secondary school students.

### 1.2 Mediating Role of Test Anxiety

Previous research findings suggest that students need to have several psychological properties for academic performance. Test anxiety, one of these psychological properties, is defined as exam‐related stress and anxiety about failure (Cassady and Johnson 2002; Chapell et al. 2005). There are several studies reporting a negative correlation between test anxiety and academic performance (Cassady and Johnson 2002; Safeer and Shah 2019). Although test anxiety can have positive effects on performance by increasing academic strategies, it can also have a debilitating effect on academic performance (Putwain 2007; Putwain et al. 2012). Findings from meta‐analyses have shown that high test anxiety is associated with academic performance (Hembree 1988; Von der Embse et al. 2018). The cognitive part of this anxiety is strongly associated with academic performance (Cassady and Johnson 2002; Hembree 1988). Therefore, this study focuses on cognitive test anxiety.

### 1.3 Mediating Role of Academic Resilience

Given its obvious benefits in terms of social, intellectual, and personal abilities in the face of challenging circumstances, there has been an increase in interest in the development of academic resilience in recent years (Allen and Eklund 2019). According to Trigueros et al. (2020), a resilient student is one who, in spite of unfavorable social or personal circumstances, performs manifestly better than anticipated. In other words, a resilient student is one who is able to respond adaptively to the demands of the environment. Research on resilience has demonstrated the connection between this concept and academic achievement as a defense against unfavorable feelings, drive, and internal well‐being. Despite these findings, academic resilience is still a novel concept, and little is known about the ways in which students' cognitive traits and choices can affect it (Cassidy 2016). There are two different studies (Hanson et al. 2003; Scales et al., 2006) that found that higher levels of resilience were strongly associated with higher grade point averages. These studies showed a positive correlation between students' test scores and almost every resilience measure.

### 1.4 Mediating Role of Self‐Efficacy

Numerous studies (Bandura 2001; Heggestad and Kanfer 2005; Vancouver et al. 2001; Zimmerman and Bandura 1994) have looked at the relationships between self‐efficacy beliefs and academic outcomes. All these researchers have identified a substantial correlation between these two variables. Academic achievement has a strong association with self‐efficacy views, according to Komarraju and Nadler's (2013) research. Self‐efficacy beliefs account for roughly 18% of the variance in academic achievement (i.e., GPA) given this positive correlation. In a similar vein, Bandura (2001) found that students' perceptions about their academic, social, and self‐regulated learning self‐efficacy predicted their academic goals and performance in the classroom. This result is consistent with the widely held opinion in the literature that there is a positive correlation between academic achievement and academic self‐beliefs. Also, some researchers found that past academic performance influenced both academic self‐efficacy and current academic performance in college students (Elias and MacDonald 2007; Hsieh et al. 2007). Since it was determined that there were few studies associating general self‐efficacy and academic achievement for secondary school students, there was a need for more studies explaining this association.

## 2 The Current Study

Academic achievement is one of the important indicators of students' long‐term mental health (Lê‐Scherban et al. 2014) and future professional success (French et al. 2015). According to certain research, smartphone addiction has been associated with lower academic performance (Amez and Baert 2020; Kates et al. 2018). The theory behind the negative causal relationship between academic achievement and smartphone addiction holds that cognitive overload, inefficiency, a lack of attention or concentration, and the inability to focus for extended periods of time can result from balancing smartphone use with work‐related activities and socializing (Dontre, 2021). The research has acknowledged the theoretically established detrimental correlation between academic achievement and smartphone addiction. In fact, the results of meta‐analysis revealed a weakly negative association (e.g., *r* = ‐0.12) between the two variables (Kates et al. 2018). This low relationship between these two variables, for which a higher relationship was theoretically expected, suggests that some other variables may mediate the relationship between these two variables. The purpose of this study is to investigate the mediating role of general self‐efficacy, academic resilience, and test anxiety in the relationship between smartphone addiction and academic achievement. For this purpose, a chain mediation model was established.

## 3 Method

### 3.1 Participants

A total of 909 seventh‐ and eighth‐grade students from seven different cities in Turkey participated in this study. After missing data and outlier analysis, the dataset was cleaned, and the final study group consisted of 828 secondary school students. Of the participant group, 51.9% (*n* = 430) were female students and 53.3% (*n* = 441) were seventh‐grade students. In addition, 33.0% (*n* = 273) of the participants stated that they spend more than 3 h a day using the Internet. 55.4% of the participants (*n* = 459) stated that they had a focusing problem in general. The inclusion criteria for the participants in the research group were as follows: (i) being a seventh or eighth grade student, (ii) having a cell phone, and (iii) obtaining their and their parents' consent to participate in the study. The authors assert that all procedures contributing to this work comply with the ethical standards of the relevant national and institutional committees on human experimentation and with the Helsinki Declaration of 1975, as revised in 2013. All procedures involving human subjects/patients were approved by the Social and Human Sciences Ethics Committee of the Akdeniz University with the number 803447. Consent forms were filled out by the students and their parents through the teachers, and data were collected through the generated website link.

### 3.2 Measures

#### 3.2.1 Personal Information Form

Various characteristics of the students, such as gender, class, time spent on studying, and internet use, were measured with this form.

#### 3.2.2 Smartphone Addiction Scale—Short Form (SAS‐SF)

The SAS‐SF (Kwon et al. 2013) is a self‐report scale with ten items and a 5‐point Likert scale (0 = largely untrue, 4 = largely true). A score between 0 and 40 can be obtained from the scale. High scores indicate high smartphone addiction. The alpha value of the original form of the scale is 0.91. The scale was adapted to Turkish culture by Akın et al. (2014). In the adaptation study, CFA was applied, and adequate model‐data fit values (RMSEA = 0.052, NFI = 0.96, CFI = 0.98, NNFI = 0.98) were obtained. The alpha value was calculated as 0.88 in the adaptation study and 0.87 in our study.

#### 3.2.3 The Academic Resilience Scale (ARS)

The ARS was developed by Martin and Marsh (2006) to measure coping with stress, distress, and pressure in the academic environment. The scale consists of six items. As a result of the confirmatory factor analysis conducted for the psychometric measurements of the scale, it was found to have good fit values (CFI = 0.98, NNFI = 0.96). The Cronbach's Alpha coefficient for the reliability of the scale was calculated as 0.89. The validity and reliability of the Turkish version of the scale was conducted by Kapıkıran (2012). As a result of the confirmatory factor analysis conducted to test the appropriateness of the unidimensional structure of the scale, the model data fit values of the scale (CFI = 0.95, NNFI = 0.91) were found to be at a good level. Cronbach's alpha coefficient for the reliability of the scale was calculated as 0.89 and 0.82 in our study.

#### 3.2.4 The Cognitive Test Anxiety Scale (CTAS)

The CTAS was developed by Cassady and Johnson (2002) and revised and finalized by Cassady and Finch (2015). The scale aims to measure cognitive indicators of test anxiety. The original scale is a 4‐point Likert scale with high validity and reliability. The scale was adapted for Turkish culture by Bozkurt et al. (2017). According to the CFA findings in the adaptation study (TLI = 0.99, RMSEA = 0.04), the scale consists of one dimension and 23 items. The alpha value was calculated as 0.93 in the adaptation study and 0.92 in our study.

#### 3.2.5 Self‐Efficacy Questionnaire for Children (SEQ‐C)

The SEQ‐C was developed by Muris (2001) to measure adolescents' social, academic, and emotional self‐efficacy. The first factor of the scale, social self‐efficacy, measures adolescents' level of awareness of peer relationships and assertiveness ability, while the second factor, academic self‐efficacy, measures adolescents' perception of their ability to realize academic expectations, to achieve academic subjects, and to manage one's own learning behavior. The last factor of the scale, emotional self‐efficacy, measures adolescents' perception of their ability to cope with negative emotions. The scale consists of 21 items in total, with seven items in each factor. As a result of the internal consistency study of the scale, the researcher found Cronbach's alpha coefficient as 0.88 for general self‐efficacy, 0.85 for social self‐efficacy, 0.88 for academic self‐efficacy, and 0.88 for emotional self‐efficacy (Muris 2001). The scale was adapted to Turkish culture by Telef and Karaca (2012). Second‐order CFA findings showed that the scale had high model‐data fit (CFI = 0.96, NNFI = 0.96, RMSEA = 0.049). In the adaptation study, the alpha values of the factors were 0.86, 0.64, 0.84, and 0.78 for general, social, academic, and emotional self‐efficacy, respectively, while they were 0.90, 0.78, 0.84, and 0.80 in our study. Both the original scale development study and the adaptation study provided evidence of a general score on the scale (e.g., high inter‐factor correlations and second‐order CFA findings). Therefore, general self‐efficacy scores were used in our study.

#### 3.2.6 Academic Achievement

The grade point average (GPA) of the most recent year of graduation was used as the academic achievement of the participant students. This data was collected through teachers. The GPA data was scaled between 0 and 100.

### 3.3 Data Analysis

**FIGURE 1:**
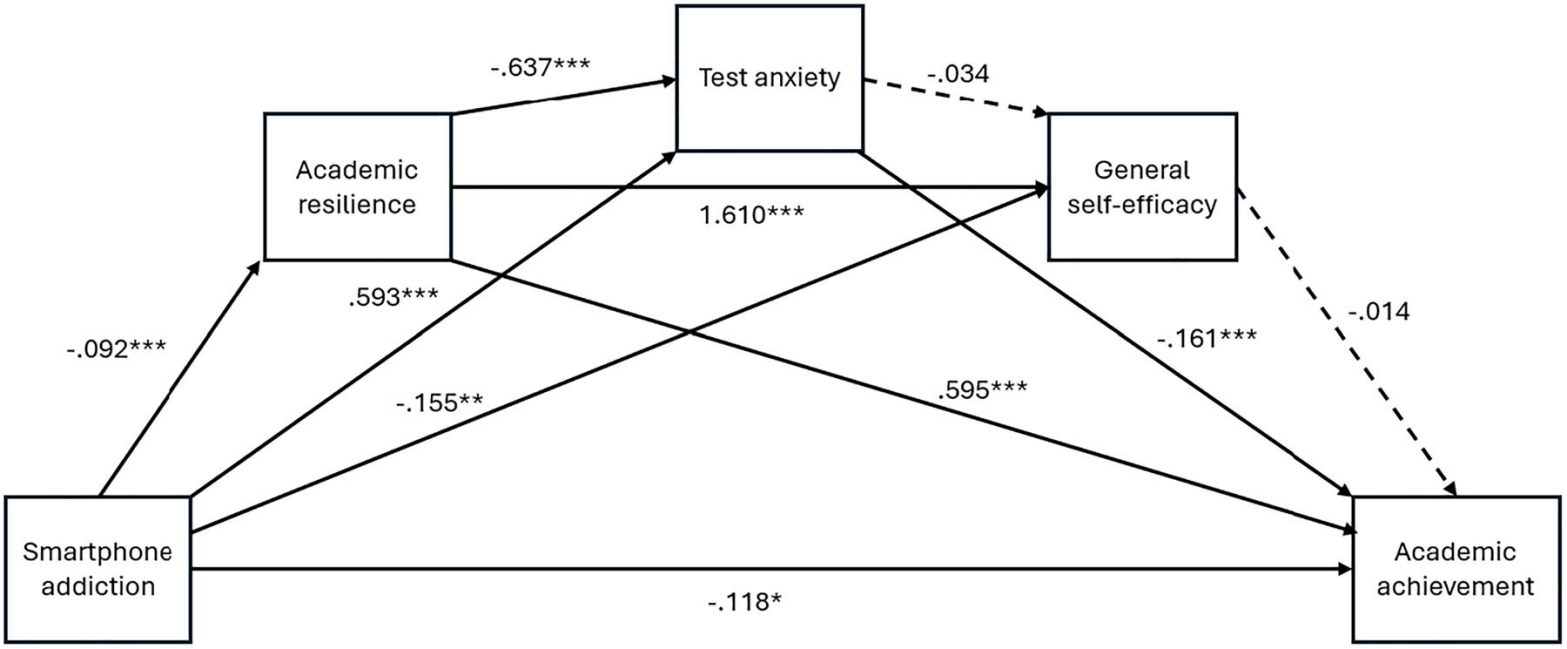
Diagram of chain mediation model.

The SPSS PROCESS macro was used for data analysis. First, Harman's single factor test (Podsakoff et al. 2003) was used to evaluate common method bias, and it was considered concerning if a single factor explained more than 50% of the general variance (Mat Roni 2014). Second, Cronbach's α values were calculated to examine the internal consistency of the measures. These values are reported in the measures subheading. Third, descriptive and correlation analyses were conducted between the variables. Fourth, to test the theoretical model proposed (Figure 1), the PROCESS macro for SPSS (Hayes 2017) was performed to examine the chain mediation model (model 6) using 10,000 bootstrapping. Due to the statistically significant correlations between the mediator variables, a chain mediation model was estimated instead of a parallel model. PROCESS calculates regression‐based ordinary least squares (Hayes 2017). 95% confidence intervals (CI) were considered significant if they did not include 0.

## 4 Results

### 4.1 Common Method Bias

Two factors had an eigenvalue greater than 1.0, according to the findings of the test for common method bias; the first factor only accounted for 31.03% of the total variance, which is less than 50%. According to the results, the bias in the common procedure was insufficient to cause significant distortion of the data.

### 4.2 Descriptive Statistics and Correlation of Variables

The mean, standard deviation, and Pearson correlation coefficients of all variables used for the study are presented in Table 1. Smartphone addiction was negatively correlated with academic resilience (*γ* = ‐0.145, *p* < 0.001), general self‐efficacy (*γ* = ‐0.186, *p* < 0.001), and academic achievement (*γ* = ‐0.189, *p* < 0.001) and positively correlated with cognitive test anxiety (*γ* = 0.416, *p* < 0.001). Academic resilience was negatively correlated with cognitive test anxiety (*γ* = ‐0.313, p < 0.001) and positively correlated with general self‐efficacy (*γ* = 0.606, *p* < 0.001) and academic achievement (*γ* = 0.318, *p* < 0.001). Negative correlations were found between cognitive test anxiety with general self‐efficacy (*γ* = ‐0.250, *p* < 0.001) and academic achievement (*γ* = ‐0.287, *p* < 0.001). A positive correlation was found between general self‐efficacy and academic achievement (*γ* = 0.200, *p* < 0.001). All correlations were statistically significant.

**TABLE 1:** Descriptive statistics and correlations among main variables (*N* = 828).

	M (SD)	SAS‐SF	ARS	CTAS	SEQ‐C	GPA
SAS‐SF	12.60 (8.71)	—				
ARS	19.88 (5.54)	−0.145*	—			
CTAS	48.42 (13.66)	0.416*	−0.313*	—		
SEQ‐C	70.39(15.27)	−0.186*	0.606*	−0.250*	—	
GPA	83.68(12.58)	−.0189*	0.318*	−0.287*	0.200*	—

* *p* < 0.001.

### 4.3 The Chain Mediating Effect of Academic Resilience, Cognitive Test Anxiety, and General Self‐Efficacy

In the theoretical model (see Figure 1), there are four different outcome variables. While three of them are mediating variables, one of them is the dependent variable. The findings of the regression models are presented in Table 2. Smartphone addiction, the only independent variable of the model, was a significant predictor of academic resilience (*b* = ‐0.09, SE = 0.02, 95% CI [‐0.14, ‐0.05]). Smartphone addiction (*b* = 0.59, SE = 0.05, 95% CI [.50, .69]) and academic resilience (*b* = ‐0.64, SE = 0.08, 95% CI [‐0.79, ‐0.49]) significantly explain another mediator variable, cognitive test anxiety. Smartphone addiction (b = ‐0.16, SE = 0.05, 95% CI [‐0.26, ‐0.05]) and academic resilience (*b* = 1.61, SE = .08, 95% CI [1.45, 1.77]) significantly predicted general self‐efficacy, while cognitive test anxiety (*b* = ‐0.03, SE = 0.04, 95% CI [‐0.10, 0.04]) did not significantly explain general self‐efficacy. In the final regression model, smartphone addiction (*b* = ‐0.12, SE = 0.05, 95% CI [‐0.22, ‐0.02]), academic resilience (*b* = 0.60, SE = 0.09, 95% CI [0.41, 0.78]), and test anxiety (*b* = ‐0.16, SE = 0.03, 95% CI [‐0.23, ‐0.09]) significantly predicted academic achievement, whereas general self‐efficacy (*b* = ‐0.01, SE = 0.03, 95% CI [‐0.08, 0.05]) did not significantly predict academic achievement. According to these findings, it is seen that the mediating variables of academic resilience and cognitive test anxiety are both predicted by the independent variable and predict the dependent variable significantly. In this case, it is possible to say that these two variables have mediating effects. However, the fact that general self‐efficacy could not predict academic achievement shows that it does not have a mediation effect. To go into more detail on this issue, the findings of seven different mediation paths were also analyzed.

**TABLE 2:** Regression results for testing chain mediation model (*N* = 828).

Variables	*b*	SE	*t*	*p*	LLCI	ULCI	*R^2^*
Outcome Variable: Academic resilience							.02
Smartphone addiction	−0.09	0.02	4.22	0.000	−0.14	−0.05	
Outcome Variable: Test anxiety							0.24
Smartphone addiction	0.59	0.05	12.31	0.000	0.50	0.69	
Academic resilience	−0.64	0.08	8.41	0.000	−0.79	−0.49	
Outcome Variable: General self‐efficacy							0.38
Smartphone addiction	−0.16	0.05	2.93	0.003	−0.26	−0.05	
Academic resilience	1.61	0.08	20.18	0.000	1.45	1.77	
Test anxiety	−0.03	0.04	0.96	0.336	−0.10	0.04	
Outcome Variable: Academic achievement							0.15
Smartphone addiction	−0.12	0.05	2.30	0.022	−0.22	−0.02	
Academic resilience	0.60	0.09	6.32	0.000	0.41	0.78	
Test anxiety	−0.16	0.03	4.73	0.000	−0.23	−0.09	
General self‐efficacy	−0.01	0.03	0.43	0.668	−0.08	0.05	

Findings for direct, indirect, and total effects are presented in Table 3 and Figure 1. The direct effect of smartphone addiction on academic achievement is statistically significant (*B* = ‐0.118, 95% CI [‐0.219, ‐0.017], *p* < 0.05). The total effect obtained when the effect of mediating variables is included is also statistically significant (*B* = ‐0.274, 95% CI [‐0.370, ‐0.177], *p* < 0.001). In this case, the inclusion of academic resilience, cognitive test anxiety, and general self‐efficacy in the model resulted in a higher regression coefficient and increased the variance explained by the model. This situation indicates a partial mediation. However, indirect effects were examined to determine which variables mediated the model. For this purpose, three of the seven different mediation paths could draw a statistically significant mediation path because the values of the unstandardized regression coefficients within the 95% confidence interval did not include zero, but four mediation paths were not statistically significant. The paths with statistically significant mediating power were (i) the mediating role of academic resilience between smartphone addiction and academic achievement (*B* = ‐0.055, 95% CI [‐0.088, ‐0.028]), (ii) the mediating role of cognitive test anxiety between smartphone addiction and academic achievement (*B* = ‐0.096, 95% CI [‐0.139, ‐0.054]), and (iii) the mediating role of academic resilience and cognitive test anxiety between smartphone addiction and academic achievement (*B* = ‐0.009, 95% CI [‐0.017, ‐0.004]). The common point of the four pathways whose mediation effect is not statistically significant is the general self‐efficacy variable. In this case, it was determined that academic resilience and cognitive test anxiety had a mediating role in predicting academic achievement of smartphone addiction, while general self‐efficacy did not have a mediating role. Therefore, it was thought that general self‐efficacy could be a potential moderator variable, and various models in which general self‐efficacy was a moderator variable were tested. However, general self‐efficacy did not show a moderator effect in any model.

**TABLE 3:** Direct effect, indirect effects and total effect (*N* = 828).

Variables	*β*	*B*	Lower	Upper
Total indirect effect	−0.107	−0.155	−0.206	−0.104
Addiction → resilience → achievement	−0.038	−0.055	−0.088	−0.028
Addiction → anxiety → achievement	−0.066	−0.096	−0.139	−0.054
Addiction → self‐efficacy → achievement	0.002	0.002	−0.008	0.014
Addiction → resilience → anxiety → achievement	−0.007	−0.009	−0.017	−0.004
Addiction → resilience → self‐efficacy → achievement	0.001	0.002	−0.007	0.013
Addiction → anxiety → self‐efficacy → achievement	0.000	0.000	−0.001	0.003
Addiction → resilience → anxiety → self‐efficacy → achievement	0.000	0.000	0.000	0.000
Total direct effect	−0.082	−0.118	−0.219	−0.017
Total effect	−0.189	−0.274	−0.370	−0.177

Abbreviations: *β* = standardized regression coefficient, *B* = unstandardized regression coefficient.

## 5 Discussion

The purpose of this study is to investigate the mediating effect of academic resilience, cognitive test anxiety, and general self‐efficacy in the association between smartphone addiction and academic achievement. For this purpose, 828 seventh‐ and eighth‐grade students in seven different cities of Turkey constituted the study group of this research. The results obtained by testing the theoretical model established with the chain mediation model are presented in this section.

First, the effect of smartphone addiction on academic achievement is negative and statistically significant. This finding is similar to previous research findings that did not work with secondary school students (Eoh et al. 2022; Kakkar et al. 2015; Lepp et al. 2014; Li et al. 2015; Samaha and Hawi 2016). In some experimental studies, it has been determined that cell phone use in the classroom has a negative effect on academic performance (Beland and Murphy 2016; Felisoni and Godoi 2018). In one of the few studies focusing on the relationship between smartphone addiction and academic achievement in middle school students, Lee and Lee (2017) found a relationship between smartphone addiction and low academic performance. However, a small portion of this study (approximately 30%) consisted of middle school students. In another study in which middle school students formed part of the research group, Seo et al. (2016) found a negative relationship between mobile phone addiction and academic performance. Considering these few studies, this association between smartphone addiction and academic achievement obtained in this study is of considerable importance.

Secondly, among the three mediator variables, the variable with the highest mediator effect according to the regression coefficients was cognitive test anxiety. There are various studies that have identified a negative relationship between test anxiety and academic achievement (Cassady and Johnson 2002; Hembree 1988; Safeer and Shah 2019; Von der Embse et al. 2018). In our study, cognitive test anxiety significantly predicted academic achievement. Test anxiety was related to lower academic performance, but low academic performance wasn't related to high test anxiety, according to a longitudinal study by Steinmayr et al. (2014) that examined the relationship between test anxiety and academic performance in middle school students. Regarding test anxiety's mediating effect in the association between academic achievement and smartphone addiction, no prior research findings were found.

The other important mediator variable in this study was academic resilience. The association between the mediator variable of academic resilience and academic achievement is positive and significant. There are various studies that determine the relationship between academic resilience and academic achievement (Cappella and Rhona 2001; Hanson et al. 2003; Scales et al. 2006). For this reason, the fact that an individual is successful and progresses in his/her academic life despite the negativities he/she experiences indicates academic resilience (Yavuz and Kutlu 2016). There are many studies on the mediating role of academic resilience (Demir 2023; Mahmoodimehr et al. 2022; Mirsadegh et al. 2021; Turan 2021). In a few studies, the mediating role of academic resilience was tested through a model in which academic achievement was the dependent variable (Choo and Prihadi 2019; Demir 2023). There is no study on the mediating role of academic resilience in the relationship between smartphone addiction and academic achievement. Academic resilience played an important mediator role in this relationship.

Cognitive test anxiety and academic resilience mediated the relationship between smartphone addiction and academic achievement. General self‐efficacy was excluded from the model since it did not have an effective mediating role. According to this finding, which indicates a partial mediation, researchers who want to investigate how much smartphone addiction affects the academic achievement of middle school students should also examine students' cognitive test anxiety and academic resilience.

### 5.1 Implications and Limitations

This study has some practical implications. First, teachers, school administrators, and central education decision‐makers should know that one of the important factors in academic achievement is smartphone addiction. It is recommended that decision‐makers should conduct some studies on reducing smartphone use in the classroom and provide training to reduce general smartphone addiction. Another important implication is that actions should be taken to reduce cognitive test anxiety and increase the academic resilience of middle school students. Since the mediating effect of general self‐efficacy was not found in this study, the mediating/moderating effect of other types of self‐efficacy, such as academic self‐efficacy, should be examined in future studies.

This study has several limitations. Although the application was carried out in seven different cities in Turkey, the findings obtained cannot be generalized to the relevant regions since these cities and students were not selected according to the Classification of Statistical Regional Units. Another limitation is that the online data collection process, even though it was carried out with the support of teachers and parents, affects the quality of the data. Thirdly, the cross‐sectional nature of the data obtained prevented the establishment of a real cause‐effect relationship.

## 6 Conclusions

With a theoretically established chain mediation model, the mediating role of cognitive test anxiety, academic resilience and general self‐efficacy on the effect of smartphone addiction on academic achievement was examined. The mediating role of cognitive test anxiety and academic resilience, in the relationship between smartphone addiction and academic achievement was revealed in this study. It is thought that these findings will guide teachers and school administrators trying to increase academic achievement.

## Author Contributions

**Hakan Koğar**: conceptualization, formal analysis, investigation, methodology, writing – original draft. **Ayfer Sayın**: conceptualization, methodology, data curation. **Güçlü Şekercioğlu**: investigation, review. **Esin Yılmaz Koğar**: investigation, writing – review and editing. **Hüseyin Kafes**: investigation, editing.

## Conflicts of Interest

The authors declare no conflicts of interest.

## Peer Review

The peer review history for this article is available at https://publons.com/publon/10.1002/brb3.70800

## Data Availability

The data set used in the study can be sent to the editor / reviewers by the correspondence author, if desired.
